# Safety and effectiveness of risdiplam in adults with spinal muscular atrophy: a systematic review

**DOI:** 10.1007/s00415-025-13557-4

**Published:** 2025-12-08

**Authors:** Paolo Alonge, Guido Urbano, Giulio Gadaleta

**Affiliations:** 1 Department of Biomedicine, Neuroscience and Advanced Diagnostics (Bi.N.D.), University of Palermo, 90127 Palermo, Italy; 2 Neuromuscular Unit, Department of Neurosciences “Rita Levi Montalcini”, University of Turin, Via Cherasco 15, 10126 Turin, Italy

**Keywords:** Adult SMA, Risdiplam, Safety, Effectiveness

## Abstract

**Background:**

Risdiplam has broadened therapeutic options for spinal muscular atrophy (SMA). While its efficacy and safety are well established in children, data in adults remain limited. This review summarizes current evidence on risdiplam use in the adult SMA population.

**Methods:**

Following PRISMA 2020 guidelines, we systematically reviewed PubMed, Scopus, and the Cochrane Library up to September 2025 for studies including risdiplam-treated adults (≥ 18 years). Outcomes were summarized across motor, bulbar, respiratory, patient-reported, safety, and adherence domains.

**Results:**

Fourteen studies (> 200 adults, mainly SMA types 2 and 3) were included. Most participants were non-ambulant and treatment naïve. Motor function was generally stable, with modest yet significant improvements on RULM, HFMSE, or MFM-32, particularly among younger/less severely affected adults. Swallowing, speech, and fatigue often improved even in advanced disease. Patient-reported outcomes consistently indicated perceived gains in quality of life across all phenotypes. Adverse events were mostly mild and transient—mainly gastrointestinal symptoms, photosensitivity, or liver enzyme elevations—with very few temporary discontinuations.

**Conclusions:**

Risdiplam shows a favorable safety profile and provides both disease stabilization and multidimensional benefits across all functional phenotypes in adults with SMA, although further longitudinal studies using standardized outcome measures are needed to clarify its long-term impact.

**Supplementary Information:**

The online version contains supplementary material available at 10.1007/s00415-025-13557-4.

## Introduction

Spinal muscular atrophy (SMA) is a genetically determined lower motor neuron disease characterized by predominantly proximal muscle weakness, often accompanied by variable degrees of respiratory and bulbar involvement [1].

Over the past several years, the advent of disease-modifying therapies (DMTs) has profoundly transformed the management of SMA, with compelling evidence of efficacy particularly in the pediatric population, where the natural course of the disease has been radically modified [2]. Increasingly, attention has also turned to adult patients, for whom the clinical response and safety profile of these treatments remain less clearly defined.

Among available DMTs, nusinersen has been the most extensively investigated in adults, with several studies suggesting potential stabilization or modest improvement in motor function [3]. In contrast, evidence on risdiplam in adults remains limited. Randomized controlled trials and real-world studies have largely focused on pediatric or adolescent cohorts. In particular, the SUNFISH trial, which supported regulatory approval of risdiplam, included only 22 participants aged 18–25 years (14 in the risdiplam and 8 in the placebo arm) [4].

Since its approval by international and national regulatory agencies beginning in 2020 [5], several small observational studies have explored the effects of risdiplam in adults with SMA. However, the current evidence base remains fragmented and preliminary. Limitations in sample size, study design, and follow-up duration continue to hinder a clear understanding of its long-term efficacy, safety, and tolerability in this population.

This systematic review aims to comprehensively synthesize the available evidence on the safety, effectiveness, and tolerability of risdiplam in adult SMA patients, providing an updated overview of its therapeutic profile in this specific population.

## Materials and methods

This systematic review was conducted following a pre-defined review protocol, in accordance with established academic standards [6]. We adhered to the Preferred Reporting Items for Systematic reviews and Meta-Analyses (PRISMA) 2020 guidelines [7].

### Eligibility criteria

Eligibility criteria were based on the population, interventions, comparators and outcomes (PICO) framework [8]:

- Population: we included studies reporting data on adult patients (≥ 18 years), with a genetically defined diagnosis of SMA. Studies with mixed populations (both adults and pediatric) were included only if data on adults could be extracted. No limits were applied regarding geographical setting.
- Intervention: we included studies with patients treated with risdiplam at any dose. Studies including patients receiving concomitant treatments (e.g., additional pharmacological treatments, physical therapy) were included only if other therapies were stable for at least 6 months before the start of risdiplam. Studies involving patients treated with various DMTs were included only if outcomes for the risdiplam cohort could be extracted.
- Comparators: control groups included placebo, best supportive care, other treatments or no treatments.
- Outcomes:
  - Efficacy: Change from baseline in motor, respiratory or bulbar function Proportion of patients with clinically meaningful improvement; ambulatory status; survival; patient-reported outcome measures (PROMs). To ensure a comprehensive review, no restrictions were placed on the specific outcome assessment tools or clinical scales used.
  - Safety and Tolerability: Incidence of Adverse Events (AEs) and Serious Adverse Events (SAEs), including AEs leading to discontinuation; Adherence to treatment.
- Time: we included all eligible studies published until the date of the final search (September 2025). No other restrictions on publication date were applied.
- Study design: English language, full-text original articles, including Randomized Controlled Trials (RCTs), prospective and retrospective observational studies, case series, and case reports were considered eligible.

### Search strategy

The literature search was conducted across three major databases: PubMed, Scopus, and the Cochrane Library. In addition, ClinicalTrials.gov was searched to identify ongoing clinical trials. To ensure completeness, relevant studies available as early online publications (e-published) on ResearchGate were also considered. We included all articles that contained the terms “*risdiplam*” AND “*adult*” in the title and/or abstract.

No formal protocol registration was performed.

### Study selection

Search results from all databases were imported into a reference management software (Mendeley Desktop. Version 1.19.8, Mendeley Ltd.). Three authors (GG, GU, and PA) independently screened all retrieved records for eligibility based on the pre-specified inclusion and exclusion criteria. Titles were initially examined, and duplicates, off-topic records, and non-English publications were excluded. Abstracts of the remaining articles were then assessed for eligibility. Each reviewer provided an inclusion (Y) or exclusion (N) judgment. Articles were advanced to the next phase if they received a majority of inclusion votes, or, in case of a tie, when the decision was balanced.

**Fig. 1:**
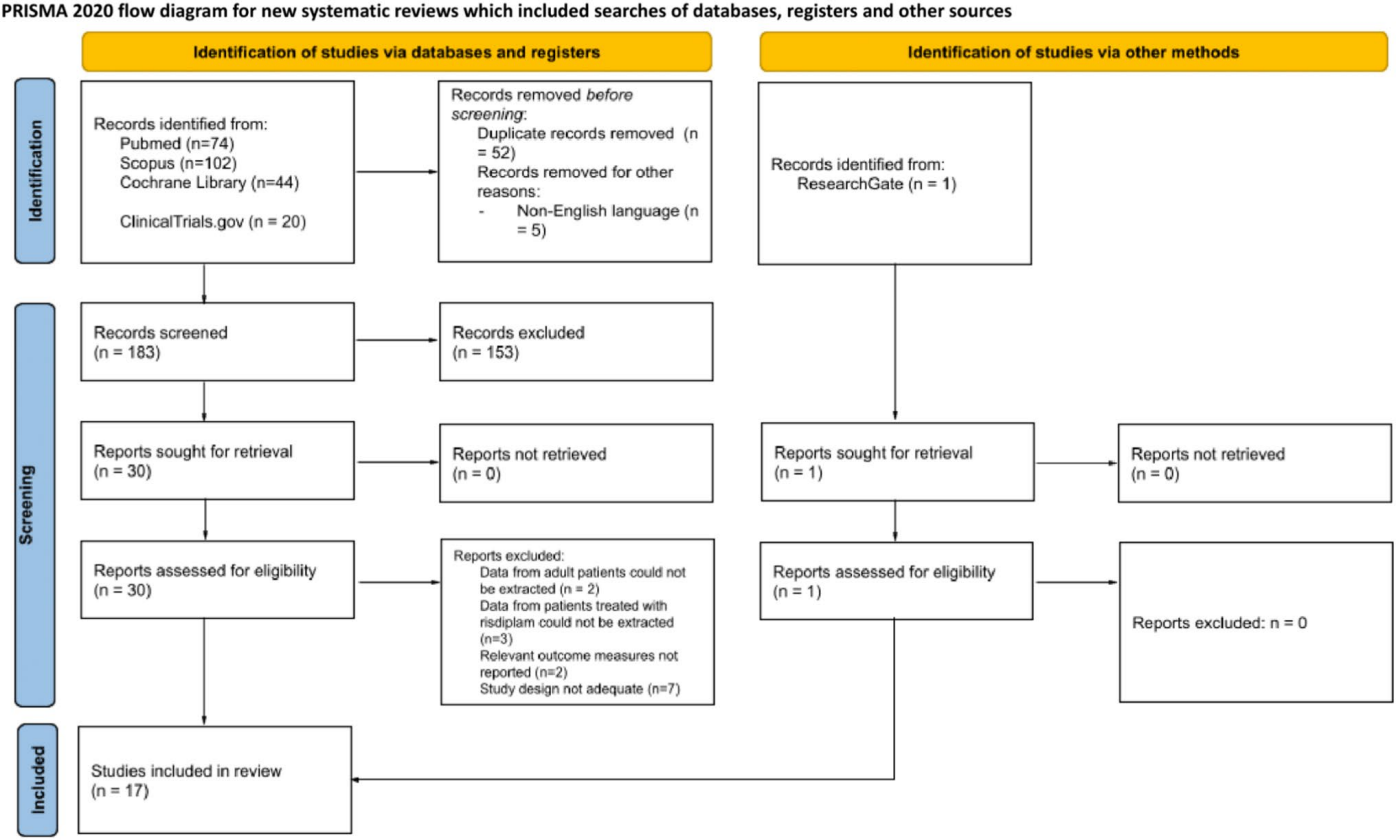
PRISMA flow diagram in the study selection process. Source: Page MJ, et al. BMJ 2021;372:n71. https://doi.org/10.1136/bmj.n71. This work is licensed under CC BY 4.0. To view a copy of this license, visit https://creativecommons.org/licenses/by/4.0/

The study selection process is summarized in a PRISMA flow diagram (Fig. 1).

### Data extraction

Data were extracted from the included studies by one reviewer (GG) using a predefined data extraction form. Two additional reviewers (PA and GU) independently verified all extracted data for accuracy. Outcomes were categorized into four domains: motor function, bulbar/respiratory function, PROMs, and safety.

The following variables were collected:

- Study characteristics: first author, year of publication, country, study design (prospective, retrospective, randomized, or case report), and follow-up duration.
- Population details: number of participants, age range or mean age, sex distribution, SMA type (I, II, III, or IV), ambulatory status (sitter/non-sitter/walker), and previous treatment exposure (treatment-naïve or switched from nusinersen).
- Motor outcomes, bulbar, swallowing, respiratory, and neurophysiological data (details of the specific outcome measures assessed are presented in the Results section).
- Patient-reported outcomes (PROs): self-reported changes in speech, chewing, swallowing, fatigue, or quality of life (e.g., INQoL, EK2, SMAIS, P-GIC, GAS).
- Safety information: type and incidence of adverse events, discontinuations, and reported tolerability.

All extracted data were cross-checked against the published tables and supplementary materials of each article to ensure accuracy and consistency. Quantitative data were reported as mean ± SD or median (interquartile range—IQR/range), as presented in the original publications.

### Risk of bias assessment

The risk of bias for the included Randomized Controlled Trials (RCTs) was assessed by three independent authors (PA, GG, and GU) using the Cochrane Risk of Bias tool 2 (RoB2) for RTCs [9]. The RoB2 is a standardized and validated instrument designed to assess the methodological quality of randomized controlled trials across five key domains: the randomization process, deviations from intended interventions, missing outcome data, measurement of the outcome, and selection of the reported result. Each domain is rated as 'low risk', 'some concerns', or 'high risk' of bias, leading to an overall judgment for each study.

Similarly, the risk of bias for the included case series studies was independently assessed by three authors (PA, GG, and GU) using the Institute of Health Economics (IHE) Quality Appraisal Checklist for Case Series [10]. The IHE checklist evaluates the methodological rigor of non-comparative studies across key domains, including selection bias (patient representativeness), measurement bias (quality of outcome assessment), attrition bias (completeness of follow-up), and reporting bias (clarity of results). Each study received a score from 0 to 40, with higher scores indicating a lower risk of bias. Any discrepancies were resolved by majority consensus.

The quality of the included studies is summarized in Table 1. Detailed quality assessments are reported in Supplementary Table 1.

**Table 1:** Methodological quality assessment of included studies

Study	Authors	Year	Total Score	Quality
Safety and efficacy of once-daily risdiplam in type 2 and non-ambulant type 3 spinal muscular atrophy (SUNFISH part 2): a phase 3, double-blind, randomised, placebo-controlled trial [4]	Mercuri et al	2022	-	Low risk of bias
Two-year efficacy and safety of risdiplam in patients with type 2 or non-ambulant type 3 spinal muscular atrophy (SMA) [11]	Oskoui et al	2024	-	Low risk of bias
Efficacy and safety of risdiplam in adults with 5q-associated spinal muscular atrophy: a nationwide observational cohort study in Austria [12]	Keritam et al	2025	35	High
Two-year Risdiplam treatment in adults with spinal muscular atrophy: improvements in motor and respiratory function, quality of life and fatigue [13]	Iterbeke et al	2025	32	High
Switching from Nusinersen to Risdiplam: A Croatian Real-World Experience on Effectiveness and Safety [14]	Belančić et al	2024	30	High
Risdiplam in Adult Patients With 5q Spinal Muscular Atrophy: A Single-Center Longitudinal Study [15]	Gavriilaki et al	2025	29	Moderate
Evaluation of risdiplam efficacy in 5q spinal muscular atrophy: A systematic comparison of electrophysiologic with clinical outcome measures [16]	Kessler et al	2024	29	Moderate
Risdiplam therapy in adults with 5q‐SMA: observational study on motor function and treatment satisfaction [17]	Bjelica et al	2024	27	Moderate
Risdiplam Real World Data—Looking Beyond Motor Neurons and Motor Function Measures [18]	Sitas et al	2024	26	Moderate
Risdiplam in non-sitter patients aged 16 years and older with 5q spinal muscular atrophy [19]	Ñungo Garzón et al	2023	25	Moderate
Risdiplam: therapeutic effects and tolerability in a small cohort of 6 adult type 2 and type 3 SMA patients [20]	Severa et al	2024	23	Moderate
Risdiplam improves subjective swallowing quality in non‐ambulatory adult patients with 5q‐spinal muscular atrophy despite advanced motor impairment [21]	Brakemeier et al	2024	22	Moderate
Risdiplam for the treatment of adults with spinal muscular atrophy: Experience of the Northern Ireland neuromuscular service [22]	McCluskey et al	2023	20	Moderate

Quality assessment was performed using the Institute of Health Economics (IHE) Quality Appraisal Checklist for Case Series Studies. Two studies (20%) achieved high quality rating, while eight studies (80%) were rated as moderate quality. No studies were classified as low quality, indicating overall acceptable methodological rigor across the included literature

### Data synthesis

Due to the heterogeneity in study populations, follow-up durations, and outcome measures, data were synthesized narratively for each outcome category (motor function, bulbar/respiratory function, PROMs, and safety). Results are also presented separately according to study design (randomized controlled trials, large cohorts, small case series, and single-case reports). Summary tables at the end of the Results section provide an overview of the key findings.

## Results

Baseline demographic and clinical characteristics of adult patients across the 14 included studies are presented in Tables 2 and 3. Most patients had SMA type 2 or 3, and the majority were non-ambulatory where ambulatory status was reported (Table 2). Most patients were treatment-naïve, with prior nusinersen exposure ranging from 0 to 35.7% across studies (Table 3). Where reported, respiratory support needs, nutritional interventions, and orthopedic complications showed some degree of variability (Table 3).

**Table 2:** Demographic characteristics and SMA classification of adults treated with risdiplam in the studied cohorts

Study (year, country)	*N*	Age (years)	SMA type 1 *n*/*N* (%)	SMA type 2 *n*/*N* (%)	SMA type 3 *n*/*N* (%)	SMA type 4 *n*/*N* (%)	Ambulatory *n*/*N* (%)	Non-ambulatory *n*/*N* (%)
Mercuri et al., 2022 (International) [4]	14	NR (range 18–25)	0/14 (0%)	NR	NR	0/14 (0%)	NR	NR
Oskoui et al., 2023 (International) [11]	10	NR (range 18–25)	0/10 (0%)	NR	NR	0/10 (0%)	NR	NR
Keritam et al., 2025 (Austria) [12]	57^°^	35.7 (28.8–43.4)^a^	2/57 (3.5%)	23/57 (40.4%)	27/57 (47.4%)	4/57 (7.0%)	13/57 (22.8%)	44/57 (77.2%)
Iterbeke et al., 2025 (Belgium) [11]	18	38.6 ± 13.5^b^	0/18 (0%)	8/18 (44.4%)	9/18 (50.0%)	1/18 (5.6%)	2/18 (11.0%)	16/18 (89%)
Belančić et al., 2024 (Croatia) [14]	7^	35.0 (21.25–44.5)ᵇ	0/7 (0%)	0/7 (0%)	7/7 (100%)	0/7 (0%)	7/7 (100%)	0/7 (0%)
Gavriilaki et al., 2025 (Greece) [15]	14	30 (18–57)ᶜ	0/14 (0%)	11/14 (78.6%)	3/14 (21.4%)	0/14 (0%)	1/14 (7.1%)	13/14 (92.9%)
Kessler et al., 2024 (Germany) [16]	18	37.8 ± 3.2^b^	0/0 (0%)	7/18 (38.9%)	11/18 (61.1%)	0/0 (0%)	2/18 (11.1%)	16/18 (88.9%)
Bjelica et al., 2024 (Germany) [17]	14*	34.5 (18–51)ᶜ	0/14 (0%)	8/14 (57.1%)	6/14 (42.9%)	0/14 (0%)	1/14 (7.1%)	13/14 (92.9%)
Sitas et al., 2024 (Croatia) [18]	31	30 (18–65)ᶜ	0/31 (0%)	15/31 (48.4%)	16/31 (51.6%)	0/31 (0%)	6/31 (19.4%)	25/31 (80.7%)
Ñungo Garzón et al., 2023 (Spain) [19]	6	35.5 (17–46)ᶜ	0/6 (0%)	6/6 (100%)	0/6 (0%)	0/6 (0%)	0/6 (0%)	6/6 (100%)
Severa et al., 2024 (France) [20]	6	41 (21–65)ᶜ	0/6 (0%)	4/6 (66.7%)	2/6 (33.3%)	0/6 (0%)	0/6 (0%)	6/6 (100%)
Brakemeier et al., 2024 (Germany) [21]	25	34.3 ± 11.3^b^	0/25 (0%)	24/25 (96.0%)	1/25 (4.0%)	0/25 (0%)	0/25 (0%)	25/25 (100%)
McCluskey et al., 2023 (N. Ireland) [22]	6	33.7 (26–44)ᶜ	0/6 (0%)	6/6 (100%)	0/6 (0%)	0/6 (0%)	0/6 (0%)	6/6 (100%)
Schön et al., 2022 (Portugal) [24]	1	27	0/1 (0%)	1/1 (100%)	0/1 (0%)	0/1 (0%)	0/1 (0%)	1/1 (100%)

Overview of demographic and clinical characteristics of adult patients with SMA treated with risdiplam throughout different studies. Data include: title and country of study, sample size (*N*), age, SMA type distribution, and ambulatory status. Unless otherwise specified, data are presented as number (*n*) and percentage (%). °Keritam included 1 patient out of 57 with unknown SMA type. ^Belančić described a mixed pediatric and adult cohort, being the 7 patients with SMA type 3a (“a” as “adult”, “p” as “pediatric”) the adult-only one. *Bjelica baseline *N* = 14 (8 SMA2/6 SMA3); motor outcomes (RULM/HFMSE) evaluable patients vary by timepoint, with RULM classification detailed on 13 patients. ^a^Median (IQR); ^b^Mean ± SD; ^c^Median (range). Abbreviations: SMA = Spinal Muscular Atrophy

**Table 3:** Clinical characteristics and previous treatment status of adults treated with risdiplam in real-world studies

Study (year, country)	*N*	NIV *n*/*N* (%)	IV/tracheostomy *n*/*N* (%)	PEG *n*/*N* (%)	Scoliosis *n*/*N* (%)	Previous spondylodesis *n*/*N* (%)	Nusinersen-experienced *n*/*N* (%)	Treatment naïve *n*/*N* (%)
Mercuri et al., 2022 (International) [4]	14	NR	NR	NR	NR	NR	0/14 (0%)	14/14 (100%)
Oskoui et al., 2023 (International) [11]	10	NR	NR	NR	NR	NR	0/10 (0%)	10/10 (100%)
Keritam et al., 2025 (Austria) [12]	57	NR	NR	NR	NR	16/57 (28.1%)	0/57 (0%)	57/57 (100%)
Iterbeke et al., 2025 (Belgium) [13]	18	2/18 (11.0%)	0/18 (0%)	0/18 (0%)	17/18 (94.4%)	12/18 (66.7%)	0/18 (0%)	18/18 (100%)
Belančić et al., 2024 (Croatia) [14]	7	0/7 (0%)	0/7 (0%)	0/7 (0%)	NR	NR	7/7 (100%)	0/7 (0%)
Gavriilaki et al., 2025 (Greece) [15]	14	2/14 (14.3%)	0/14 (0%)	0/14 (0%)	NR	NR	1/14 (7.1%)	13/14 (92.9%)
Kessler et al., 2024 (Germany) [16]	18	NR	NR	NR	NR	10/18 (55.6%)	0/18 (0%)	18/18 (100%)
Bjelica et al., 2024 (Germany) [17]	14	5/14 (35.7%)	0/14 (0%)	0/14 (0%)	12/14 (85.7%)	NR	5/14 (35.7%)	9/14 (64.3%)
Sitas et al., 2024 (Croatia) [18]	31	NR	NR	NR	NR	12/31 (38.7%)	0/31 (0%)	31/31 (100%)
Ñungo Garzón et al., 2023 (Spain) [19]	6	4/6 (66.7%)	0/6 (0%)	0/6 (0%)	4/6 (67.0%)	NR	2/6 (33.3%)	4/6 (66.7%)
Severa et al., 2024 (France) [20]	6	NR	NR	NR	NR	NR	2/6 (33.3%)	4/6 (66.7%)
Brakemeier et al., 2024 (Germany) [21]	25	10/25 (40.0%)	0/25 (0%)	0/25 (0%)	NR	14/25 (56.0%)	0/25 (0%)	25/25 (100%)
McCluskey et al., 2023 (N. Ireland) [22]	6	3/6 (50.0%)	1/6 (16.7%)	NR	6/6 (100%)	4/6 (66.7%)	0/6 (0%)	6/6 (100%)
Schön et al., 2022 (Portugal) [24]	1	1/1 (100%)	0/1 (0%)	NR	NR	1/1 (100%)	0/1 (0%)	1/1 (100%)

Overview of clinical characteristics and previous treatment status of adults treated with risdiplam throughout the studies. Data include: title and country of study, sample size (*N*), ventilatory and nutritional support, scoliosis and prior spondylodesis, as well as previous exposure to nusinersen. Unless otherwise specified, data are presented as number (*n*) and percentage (%). Abbreviations: IV, Invasive Ventilation; NIV, Non-Invasive Ventilation; NR, Not Reported; PEG, Percutaneous Endoscopic Gastrostomy

Clinical outcomes, including motor, bulbar, respiratory, and patient-reported measures, are detailed in Tables 4 and 5 and are summarized in the following paragraphs.

**Table 4:** Summary of motor and bulbar function outcomes in adult SMA patients treated with risdiplam across studies

Study (year, country)	Follow-up	Motor scales	Motor outcomes	Bulbar scales	Bulbar outcomes
Mercuri et al., 2022 (International) [4]	12 months	HFMSE, RULM, MFM-32	Non-significant mean gain in RULM (estimate + 1.74), with no signal on HFMSE; no published data on MFM*	NR	NR
Oskoui et al., 2023 (International) [11]	24 months	HFMSE, RULM, MFM-32	Mean + 2.2 points on HFMSE and + 2.8 points on RULM. No published data on MFM*	NR	NR
Keritam et al., 2025 (Austria) [12]	Up to median 23 months (range 12–25 months)	HFMSE, RULM, ALSFRS-R	HFMSE: + 1.73 (*n* = 26) (*p* < 0.01) and RULM + 2.76 (*n* = 34) (*p* < 0.01); ALSFRS-R + 1.36 (*n* = 28) (*p* < 0.01); 6MWT + 47.6 (*p* = 0.47) (*n* = 7)	NR	NR
Iterbeke et al., 2025 (Belgium) [13]	24 months	RULM, MFM-32, Hand grip/pinch	RULM: + 1 point at 12 m (*p* = 0.02), stable at 24 m; 29% improved ≥ 2 points, 12% declined at 24 m. MFM-32: + 2.3% at 12 m (*p* < 0.01), + 2.5% at 24 m (*p* < 0.01), particularly in D3 domain → 47% improved ≥ 3 points; 8/18 achieved CMI. MRC strength: stable. Hand grip/pinch: mostly unmeasurable, no changes	FOIS, NdSSS, SSQ	FOIS and NdSSS: small non-significant improvements. SSQ: significant improvement at 12 m and 24 m (mean − 3.4, *p* = 0.01; mean − 5.6 points, *p* < 0.01)
Belančić et al., 2024 (Croatia) [14]	12 months	RHS	RHS not significantly increased (+ 0.8 from baseline, *p* = 0.5)	NR	NR
Gavriilaki et al., 2025 (Greece) [15]	Median 28.5 months (range 6–30)	HFMSE, RULM, MFM-32, 6MWT, MRC sum score	Mean Δ: HFMSE + 1.5 (*p* = 0.02), RULM + 1.6 (*p* < 0.01), MFM-32 + 2.7 (*p* < 0.01). MRC UL + 3 (*p* = 0.02), MRC LL + 1.7 (*p* = 0.05). 82% reached CMI on ≥ 1 scale	NR	NR
Kessler et al., 2024 (Germany) [16]	10 months	HFMSE, RULM	HFMSE increased + 1.17 ± 0.39 (*p* = 0.014); CHOP INTEND improved, RULM not significant	NR	NR
Bjelica et al., 2024 (Germany) [17]	Up to 20 months (50% reached 20-month follow-up)	HFMSE, RULM	HFMSE: 12/13 (92.3%) stable; 0 CMI. RULM: 4/13 achieved CMI (≥ 2 points); others stable/worsened; limited group trend	NR	NR
Sitas et al., 2024 (Croatia) [18]	> 12 months (12/31, 39% treated for 29–31 months)	HFMSE, RULM, RHS, 6MWT	CMI on ≥ 1 motor scale achieved in 7 patients (22.6%), all SMA 3. RHS (SMA3 stronger patients, *n* = 6): 3/6 had meaningful improvement (mean + 2.3 pts, *p* = 0.03). 6MWT (*n* = 4): mean change − 9 m (*p* = 0.62). Overall: majority stable, 22.6% improved, ~ 16% worsened	JFLS, INQoL	9/15 SMA2 (60%) reported improvement in swallowing, speech, chewing (mean − 2.1 pts, *p* = 0.02); ½ SMA3 (50%) with dysphagia reported improvement in swallowing
Ñungo Garzón et al., 2023 (Spain) [19]	12 months	RULM, EK2, ALSFRS-R	All 6 improved on ≥ 1 scale; 2/6 (33.3%) achieved CMI in RULM (> 2 pts); EK2 improved (mean − 4 pts, *p* = 0.03) in all patients; ALSFRS-R (*n* = 6): Mean change + 3.8 points (*p* = 0.04)	EK2 (bulbar items), BMI	Speech/swallowing items improved (5/6, 83.3%); BMI > 5% increase in 2/6 (33.3%)
Severa et al., 2024 (France) [20]	7–27 months	HFMSE, MFM-32	MFM-32 improved in 4/6 (mean + 2.16% to + 7.29%); HFMSE largely unchanged	Patient-reported (voice, chewing, breath fatigue)	Improved voice intelligibility (*n* = 5, 83%), cough strength and fatigue (*n* = 4, 67%), chewing and swallowing (*n* = 2, 33%)
Brakemeier et al., 2024 (Germany) [21]	12 months	HFMSE, RULM	HFMSE: baseline mean 1.96 (0–11) → no significant change at 12 m (2.26). RULM: baseline mean 8.8 (0–16) → slight but statistically significant improvement at 12 m (9.16, + 0.3 points, *p* = 0.018). Overall: motor function essentially stable, tiny gains but not clinically meaningful	SSQ, b-ALSFRS-R	SSQ: Baseline mean 19.5 (0–65) → 15.6 at 12 m (*p* = 0.011). 83% of patients reported improved swallowing. Bulbar ALSFRS-R subscore: Baseline 10.1 → 10.4 at 12 m (trend, not significant)
McCluskey et al., 2023 (N. Ireland) [22]	9 months	RULM, EK2	RULM reported unchanged (+ 3.2 but *p* = 0.06), while EK2 improved (mean − 4.5 over 9 months, *p* < 0.01)	Patient-reported (speech)	Better speech quality reported by all patients
Schön et al., 2022 (Portugal) [24]	12 months	HFMSE, RULM, MFM-32	RULM + 2, HFMSE + 3, and MFM32: D2 domain + 18.9 and D3 domain + 4.8 (no *p*-values)	NR	NR

Motor and bulbar function outcomes in adult patients treated with risdiplam across studies. Studies are ordered by study design (RCTs first) followed by methodological quality score. *Data not yet published at time of manuscript preparation. Abbreviations: 6MWT, 6-min walk test; ALSFRS-R, Amyotrophic Lateral Sclerosis Functional Rating Scale-Revised; b-ALSFRS-R, bulbar subscale of ALSFRS-R; BMI, Body Mass Index; CHOP INTEND, Children's Hospital of Philadelphia Infant Test of Neuromuscular Disorders; CMI, Clinically Meaningful Improvement; D2, MFM-32 domain 2 (axial and proximal motor function); D3, MFM-32 domain 3 (distal motor function); EK2, Egen Klassifikation scale version 2; FOIS, Functional Oral Intake Scale; HFMSE, Hammersmith Functional Motor Scale Expanded; JFLS, Jaw Functional Limitation Scale; LL, lower limbs; MFM-32, Motor Function Measure-32; MRC, Medical Research Council; N. Ireland, Northern Ireland; NdSSS, Neuromuscular Disease Swallowing Status Scale; NR, not reported; RCT, Randomized Controlled Trial; RHS, Revised Hammersmith Scale; RULM, Revised Upper Limb Module; SMA, Spinal Muscular Atrophy; SSQ, Sydney Swallow Questionnaire; UL, upper limbs

**Table 5:** Patient-reported and respiratory outcomes in adults with SMA treated with risdiplam across studies

Study (year, country)	Follow-up	PRO scales	PRO outcomes	Respiratory scales	Respiratory outcomes
Mercuri et al., 2022 (International) [4]	12 months	NR	NR	NR	NR
Oskoui et al., 2023 (International) [11]	24 months	NR	NR	NR	NR
Keritam et al., 2025 (Austria) [12]	Up to median 23 months (range 12–25 months)	NR	NR	NR	NR
Iterbeke et al., 2025 (Belgium) [13]	24 months	FSS, SMAIS, SF-36	FSS: Δ − 4.7 (12 months, *p* = 0.03); Δ − 5.8 (24 months, *p* < 0.01). SMAIS: Δ + 0.4 (12 months, *p* = 0.4); Δ + 1.0 (24 months, *p* = 0.3). SF-36: general improvement, more significantly in 'General Health' (+ 8.8%, *p* = 0.04 at 24 months) and 'Health Change' (+ 19.4%, p = 0.02 at 12 months; + 17.6%, *p* = 0.02 at 24 months)	FVC (%), PEF	Δ FVC increased + 0.6% (12 months) and + 1.3% (24 months) with no statistical significance. Δ PEF: + 4.2% (12 months, *p* = 0.2); + 6.9% (24 months; *p* = 0.03)
Belančić et al., 2024 (Croatia) [14]	12 months	NR	NR	Respiratory support need or switch from BiPAP to MV	None
Gavriilaki et al., 2025 (Greece) [15]	Median 28.5 months (range 6–30)	NR	NR	FVC (% pred)	No significant changes with baseline assessments (mean difference 1 [95% CI − 2.6–4.6], *p* = 0.7). Six SMA 2 patients (75%) had a FVC% decrease
Kessler et al., 2024 (Germany) [16]	10 months	NR	NR	NR	NR
Bjelica et al., 2024 (Germany) [17]	Up to 20 months (50% reached 20-month follow-up)	TSQM	All were at least "somewhat satisfied" at month 8; only one patient was very dissatisfied at month 12. No correlations with motor scale changes or adverse events	NR	NR
Sitas et al., 2024 (Croatia) [18]	> 12 months (12/31, 39% treated for 29–31 months)	INQoL	No worsening in QoL; of those who complained, improvement described in: 22/26 (85%) fatigue; 21/31 (68%) muscle weakness; 4/7 (57%) pain. All patients described beneficial effects; 3 reported concomitant harmful effects	NR	NR
Ñungo Garzón et al., 2023 (Spain) [19]	12 months	GAS, P-GIC	4/6 patients achieved a goal at GAS; 5/6 patients reported improvements at P-GIC	FVC (% pred)	No 'significant' change (> 5%) in any patient
Severa et al., 2024 (France) [20]	7–27 months	Descriptive PROs	Most improved items were hand dexterity and strength, speech and voice, and breath fatigue	NR	NR
Brakemeier et al., 2024 (Germany) [21]	12 months	NR	NR	NR	NR
McCluskey et al., 2023 (N. Ireland) [22]	9 months	QOLM, ESS	Significant improvement in QOLM (mean change + 10.7, *p* = 0.27); no significant changes in Epworth (mean change − 0.5; *p* = 0.713)	FEV1 (% pred), FVC (% pred)	No significant changes in FEV1 and FVC (+ 1.1%, *p* = 0.521; + 3.5%, *p* = 0.64)
Schön et al., 2022 (Portugal) [24]	12 months	NR	NR	FVC (ml), MIP (cmH₂O), MEP (cmH₂O)	Improvement in respiratory outcome measures

Patient-reported outcomes and respiratory function in adult patients treated with risdiplam across studies. Abbreviations**:** BiPAP, Bilevel Positive Airway Pressure; CI, Confidence Interval; ESS, Epworth Sleepiness Scale; FEV1, Forced Expiratory Volume in 1 s; FSS, Fatigue Severity Scale; FVC, Forced Vital Capacity; GAS, Goal Attainment Scale; INQoL, Individualized Neuromuscular Quality of Life; MEP, Maximal Expiratory Pressure; MIP, Maximal Inspiratory Pressure; MV, mechanical ventilation; N. Ireland, Northern Ireland; NR, not reported; P-GIC, Patient Global Impression of Change; PEF, Peak Expiratory Flow; pred, predicted; PRO, Patient-Reported Outcome; QoL, quality of life; QOLM, Quality of Life Measure; RCT, Randomized Controlled Trial; SF-36, Short Form-36 Health Survey; SMA, Spinal Muscular Atrophy; SMAIS, Spinal Muscular Atrophy Independence Scale = TSQM, Treatment Satisfaction Questionnaire for Medication

### Motor outcomes

Motor outcomes in adults were evaluated across several validated scales capturing upper-limb function, gross motor performance, multidomain motor abilities, ambulation, global function, and strength.

#### Upper limb performance (RULM)

The Revised Upper Limb Module (RULM) [23] was the most frequently used motor scale (*n* = 12), and across studies it consistently showed modest but clinically relevant improvements in a subset of adults, with overall stability in the remainder. In SUNFISH Part 2, adults gained + 1.74 points at 12 months, although this was not statistically significant [4]. In the open-label extension, improvements increased to + 2.1 and + 2.8 points at 18 and 24 months, respectively [11]. Observational studies reported changes ranging from + 0.3 (*p* = 0.018) in a non-ambulant cohort (19) to + 3.2 points (*p* = 0.06) at 9 months in a small series [22]. Larger cohorts showed significant mean gains, including + 2.76 (*p* < 0.01) at ~ 23 months [12], + 1 point at 12 months (*p* = 0.02) with stabilization thereafter [13], and + 1.6 (*p* < 0.01) at 28.5 months [15]. CMI thresholds (≥ 2 points) were achieved in 29.0% of adults in one study [13] and in 30.8% in another case series [17]. Improvements were also observed in non-sitters: 2/6 gained ≥ 2 RULM points [19]. A single case report described a + 2-point gain at 12 months [24].

#### Gross motor function (HFMSE, RHS, MFM-32, and 6MWT)

The Hammersmith Functional Motor Scale–Expanded (HFMSE) [25] was reported in 10 studies. In adults, HFMSE typically reflected stability or small improvements, consistent with expected floor effects in more impaired individuals. In SUNFISH Part 2, HFMSE did not change over 12 months [4], but in the extension phase adults showed gradual improvement, reaching + 1.4 at 18 months and + 2.2 at 24 months [11]. Observational studies reported significant changes, including + 1.73 (*p* < 0.01) [10], + 1.5 (*p* = 0.02) [15], and + 1.17 ± 0.39 (*p* = 0.014) [16]. Several series documented stability without decline [15, 19]. In a non-sitter cohort, HFMSE remained unchanged despite improvements in other scales [20].

The Revised Hammersmith Scale (RHS) [26] was used in stronger adults. Improvements were observed in stronger type 3 adults, with mean + 2.3 points at 12 months (*p* = 0.03) [18]. In a nusinersen-experienced cohort, RHS changes were smaller (+ 0.8, *p* = 0.5) [14].

The Motor Function Measure (MFM-32) [27], included in six studies, consistently captured subtle multidomain gains even when other scales remained stable.

Improvements ranged from + 2.3 to + 2.5% at 12–24 months (both *p* < 0.01) [13] and + 2.7 at 28.5 months (*p* < 0.01) [15]. Smaller cohorts showed gains between + 2.16 and + 7.29% [20]. Nearly half of adults (47.0%) improved ≥ 3 points in one 24-month study [13]. A case report documented improvements in MFM-32 domains D2 (+ 18.9) and D3 (+ 4.8) [24].

The 6-Minute Walk Test (6MWT), used only in ambulatory individuals [28], demonstrated maintenance of endurance, contrasting with expected natural-history decline. Distance walked remained stable over 23 months (+ 47.6 m, *p* = 0.47) [12] and unchanged over 12 months in another ambulatory cohort [18].

#### Global functional response (ALSFRS-R and EK2)

Amyotrophic Lateral Sclerosis Functional Rating Scale–Revised (ALSFRS-R) [29] and Egen Klassifikation 2 (EK2) [30] captured broader aspects of daily function and seemed informative in more impaired adults. ALSFRS-R improved significantly in a large cohort (+ 1.36, *p* < 0.01) [12] and in non-sitters (+ 3.8, *p* = 0.04) [19]. EK2 revealed meaningful improvements: − 4.5 (*p* < 0.01) at 9 months [22] and − 4 (*p* = 0.03) in non-sitters [19]. EK2 bulbar items also improved significantly [19].

#### Other measures (MRC and dynamometry)

Strength was reported infrequently [31, 32] and showed small but measurable gains in selected cohorts. One study documented improvements in upper-limb Medical Research Council (MRC) scale (+ 3, *p* = 0.02) and lower-limb MRC (+ 1.7, *p* = 0.05) over 28.5 months [15]. Dynamometry did not show any change [13].

### Bulbar outcomes

Bulbar outcomes were assessed through a combination of quantitative scales and patient-reported data.

#### Swallowing function

Swallowing ability consistently improved across studies, both quantitatively and subjectively. The Sydney Swallow Questionnaire (SSQ) [33] showed significant reductions over time, decreasing from 19.5 to 15.6 at 12 months (*p* = 0.011), with 83% of adults reporting easier swallowing [21]. A second long-term cohort confirmed these findings, reporting significant SSQ reductions at both 12 months (− 3.4, *p* = 0.01) and 24 months (− 5.6, *p* < 0.01) [13]. Accordingly, FOIS [34] and NdSSS [35] demonstrated positive, although non-significant, trends consistent with improved oral intake and swallowing safety [13]. EK2 bulbar items improved significantly in non-sitters (− 4, *p* = 0.03) [19], indicating better feeding and swallowing-related function. Two adults in this cohort also achieved > 5% BMI gain, suggesting nutritionally meaningful improvement [19].

#### Oro-facial and chewing function

Oro-facial performance, evaluated with the Jaw Functional Limitation Scale (JFLS) [36], also improved significantly in adults with baseline chewing or facial-motor limitations (− 2.1, *p* = 0.02) [18]. Subjective reports confirmed these findings, describing reduced chewing fatigue, greater endurance during meals and easier execution of oral-motor tasks [20, 22].

#### Speech and voice

Speech- and voice-related symptoms improved across several series, although no formal speech assessment tool was applied. Adults frequently described clearer articulation, reduced voice fatigue, and improved breath support during speech [20, 22]. These changes were often accompanied by reduced coughing fatigue and improved airway control during phonation [20, 22].

### Respiratory outcomes

Respiratory outcomes were assessed using spirometric measures and ventilation status.

#### Standard spirometry (FVC, PEF, FEV1)

Standard spirometry, comprising Forced Vital Capacity (FVC), Peak Expiratory Flow (PEF), and Forced Expiratory Volume in 1 s (FEV1), was the most commonly used respiratory assessment [37].

FVC remained stable across all adult cohorts. In a 24-month study, FVC showed minimal change at 12 months (+ 0.6%, *p* = 0.7) and 24 months (+ 1.3%, *p* = 0.3) [13]. A 28.5-month dataset confirmed overall stability, with declines observed only among adults with SMA type 2 (75.0% of the subgroup) [15]. A 9-month cohort recorded a small, non-significant increase (+ 3.5%, *p* = 0.064) [20]. In a non-sitter series, FVC remained unchanged over 12 months [19].

PEF was the only spirometric parameter showing consistent improvement, with significant increases at both 12 months (+ 4.2%, *p* = 0.03) and 24 months (+ 6.9%, *p* = 0.03) [13], suggesting enhanced expiratory airflow.

FEV1 also remained stable, showing a small, non-significant improvement (+ 1.1%, *p* = 0.521) over 9 months in a cohort with advanced respiratory involvement [22].

#### Respiratory muscle pressures (MIP/MEP)

Respiratory muscle strength was rarely assessed [38]. In the single case documenting these measures, both Maximum Inspiratory Pressure (MIP) and Maximum Expiratory Pressure (MEP) improved within 6 months and continued to rise at 12 months [24], suggesting possible reinforcement of respiratory muscle performance in severely affected adults.

#### Ventilation requirements (NIV/IV)

Ventilation needs remained unchanged. Adults using Non-Invasive Ventilation (NIV) or intermittent Invasive Ventilation (IV) at baseline maintained their pre-treatment support levels across follow-up periods of 9–12 months [15, 19, 22]. No escalation or new initiation of ventilatory support was reported.

### Patient-reported outcomes (PROs)

Patient-reported outcomes captured key domains of patients’ lived experience, including fatigue, perceived physical capacity, psychosocial wellbeing, and treatment satisfaction.

#### Fatigue and perceived motor function

Functional well-being improved across all studies incorporating fatigue and patient-perceived functional measures. The Fatigue Severity Scale (FSS) [39] showed significant reductions at both 12 months (− 4.7, *p* = 0.03) and 24 months (− 5.8, *p* < 0.01) [13], indicating a sustained decrease in fatigue burden. The fatigue domain of the Individualized Neuromuscular Quality of Life (INQoL) [40] aligned with this pattern, with 85% of adults reporting improvement [18]. Qualitative reports further described greater stamina for daily activities and reduced end-of-day exhaustion [20, 22].

Perceived physical function improved in parallel. According to the Patient’s Global Impression of Change (PGIC), 5/6 adults reported overall improvement, and 4/6 achieved at least one personal goal on the Goal Attainment Scale (GAS) [19, 41]. The INQoL [40] “weakness” domain improved in 68.0% of adults [18], with participants describing better arm use, easier transfers, and improved tolerance of upright positions [20].

#### Quality of life and psychosocial wellbeing

Health-related quality of life improved across multiple instruments. The Short Form–36 (SF-36) [42] revealed significant gains in “General Health” (+ 8.8%, *p* = 0.04) and marked improvements in the “Health Change” domain at 12 months (+ 19.4%, *p* = 0.02) and 24 months (+ 17.6%, *p* = 0.02) [13]. INQoL [40] also indicated broader symptom relief, including improvement in pain in 57% of adults and enhanced emotional wellbeing [18]. Findings from the Quality of Life Measure for People with Slowly Progressive Neuromuscular Disorders (QOLM) [39] supported this trend (mean increase + 10.7) [22].

Across these instruments, adults described feeling more confident in everyday activities, less frustrated by functional limitations, and more positive in their day-to-day outlook [18, 20].

#### Treatment satisfaction and other patient-reported domains

Treatment satisfaction was uniformly high. On the Treatment Satisfaction Questionnaire for Medication (TSQM) [43], nearly all adults rated their experience positively after 12 months, with only one individual reporting dissatisfaction [17]. Satisfaction did not correlate with motor scale changes or adverse events, suggesting that perceived benefits extended beyond objective measures.

Daytime sleepiness, assessed with the Epworth Sleepiness Scale (ESS) [44], remained stable (*p* = 0.713) [22], indicating that improvements in fatigue, wellbeing and perceived function were not attributable to changes in alertness.

### Safety

Safety findings are summarized in Table 6 and supported by observational cohorts, case series, and pharmacovigilance analyses. Across adult populations, risdiplam demonstrated a favorable and generally manageable safety profile, with most adverse events classified as mild to moderate.

**Table 6:** Safety profile and adverse events in adults treated with risdiplam

Study (Year, Country)	*N*	Follow-up	SMA type	Patients with AE *n* (%)	Number of AE	Type of AE	Number of SAE	Type of SAE	Discontinuation *n* (%)
Mercuri et al., 2022 (International) [4]	14	12 months	NR	NR	NR	NR	NR	NR	NR
Oskoui et al., 2023 (International) [11]	10	24 months	NR	NR	NR	NR	NR	NR	NR
Iterbeke et al., 2025 (Belgium) [13]	18	24 months	SMA 2: 8 (44.0%); SMA 3: 9 (50.0%); SMA 4: 1 (6.0%)	5/18 (27.7%)	8	Abdominal pain (2), diarrhea (1), mouth aphthae (1), muscle pain (1), menorrhagia (1), arthralgia (1), bladder infection (1)	1	SAE-NT: aspiration pneumonia and death (1/18, 6%)	2/18 (12%), then restarted
Belančić et al., 2024 (Croatia) [14]	7	12 months	SMA 3: 7 (100%)	NR	NR	NR	NR	NR	NR
Gavriilaki et al., 2025 (Greece) [15]	14	30 months	SMA 2: 11 (78.6%); SMA 3: 3 (21.4%)	6/14 (42.8%)	7	Gastrointestinal (constipation/diarrhea/nausea) (4, 28.6%); numbness (1, 7.1%); headache (1, 7.1%); olfactory hallucination (1, 7.1%)	0	None	0
Kessler et al., 2024 (Germany) [16]	18	10 months	SMA 2: 7 (38.9%); SMA 3: 11 (61.1%)	NR	NR	NR	NR	NR	NR
Bjelica et al., 2024 (Germany) [17]	14	Up to 20 months	SMA 2: 8 (57.1%); SMA 3: 6 (42.9%)	8/14 (57.1%)	14	Liver transaminases elevation (5, 35.7%); skin rash/sensitivity (3, 21.4%); diarrhea (3, 21.4%); aphthous ulcer (2, 14.3%); abdominal pain (2, 14.3%); constipation, otitis media, gingivitis, SARS-CoV-2 infection (1 each, 7.1%)	0	None	0
Sitas et al., 2024 (Croatia) [18]	31	> 12 months	SMA 2: 15 (48.4%); SMA 3: 16 (51.6%)	8/31 (25.8%)	12	Headache (3, 37.5%); aphthous ulcers (2, 25%); nausea, Ramsay Hunt syndrome, hyperuricemia, macrohematuria, anemia, feeling bloated, insomnia (1 each, 12.5%)	1	1 death	2/31 (6.5%), then restarted
Ñungo Garzón et al., 2023 (Spain) [19]	6	12 months	SMA 2: 6 (100%)	1/6 (16.7%)	2	Gastrointestinal symptoms and headache (1, 16.7%)	0	None	1/6 (16.7%), then restarted
Severa et al., 2024 (France) [20]	6	7–27 months	SMA 2: 4 (66.7%); SMA 3: 2 (33.3%)	NR	17	Photosensitivity (4, 67%); diarrhea (4, 67%); transaminase elevation (3, 50%); vomiting, cough, headache, dry skin (1 each)	0	None	0
Brakemeier et al., 2024 (Germany) [21]	25	12 months	SMA 2: 24 (96.0%); SMA 3: 1 (4.0%)	NR	NR	NR	NR	NR	NR
McCluskey et al., 2023 (N. Ireland) [22]	6	9 months	SMA 2: 6 (100%)	6/6 (100%)	9	Skin sensitivity to sunlight (6, 100%); diarrhea/constipation (1, 16.7%); nephrolithiasis (1, 16.7%); elevated transaminase (1, 16.7%)	0	None	1/6 (16.7%), then restarted
Keritam et al., 2025 (Austria) [12]	57	Up to median 23 months	SMA 1: 2 (3.5%); SMA 2: 23 (40.4%); SMA 3: 27 (47.4%); SMA 4: 4 (7.0%)	8/57 (14.0%)	12	Dry or sensitive skin (3, 37.5%); gastrointestinal (2, 25%); elevated amylase and lipase, nausea, headache, concentrated urine, bone fracture, acute cardiac event, infection (1 each, 12.5%)	NR*	NR*	NR
Schön et al., 2022 (Portugal) [24]	1	12 months	SMA 2: 1 (100%)	NR	NR	NR	NR	NR	NR
Yu & Liu, 2024 (China)ᵃ [45]	NR	Since 2020–2024	NR	NR	406	Age 18–45 years: general disorders and administration site conditions (139); gastrointestinal disorders (104); infections and infestations (88). Age 45–65 years: gastrointestinal disorders (39); general disorders and administration site conditions (37); infections and infestations (27). Age > 65 years: infections and infestations (9); general disorders and administration site conditions (9); gastrointestinal disorders (6)	NR	NR	NR

Safety profile and adverse events in adult patients treated with risdiplam studies. *Keritam et al. reported that SAE data were not available in their study. ᵃYu & Liu, 2024 represents pharmacovigilance data from the FDA Adverse Event Reporting System (FAERS) database and is not included in the studies quality assessment. Abbreviations: AE, adverse event; N. Ireland, Northern Ireland; NR, not reported; SAE, serious adverse event; SAE-NT, serious adverse event not treatment-related; SARS-CoV-2, severe acute respiratory syndrome coronavirus 2; SMA, Spinal Muscular Atrophy

Adverse events occurred in 14.0% to 57.1% of adults, with the most frequently reported categories including gastrointestinal symptoms (constipation, diarrhea, nausea), photosensitivity or skin sensitivity, and elevations in liver transaminases [12, 13, 15, 17, 18, 20, 22]. Liver transaminase elevation was particularly common in some cohorts, reaching 35.7% of adults [17]. These abnormalities were typically transient and manageable without permanent discontinuation. Notably, one small cohort (*n* = 6) reported photosensitivity in all participants [22], but this isolated finding was not reproduced in larger samples and should be interpreted with caution given the limited sample size. Pharmacovigilance data from the FDA Adverse Event Reporting System (FAERS) corroborated these findings, with gastrointestinal disorders, general-condition symptoms, and infections representing the most frequently reported categories in treated adults [45].

Temporary treatment interruptions occurred in a minority of individuals—usually secondary to gastrointestinal complaints or photosensitivity [13, 18, 22]—and were brief, with the longest suspension lasting approximately 1.5 months [19]. Permanent discontinuation was rare across all studies.

Two deaths were reported: one associated with aspiration pneumonia [13], and one attributed to comorbid conditions in a mixed SMA2/SMA3 cohort [18]. Both were deemed unrelated to risdiplam. No treatment-related (SAEs) were identified.

### Adherence

Adherence to daily oral risdiplam was consistently high across clinical trials, observational cohorts, and real-world data sources.

In SUNFISH, no adult discontinued treatment due to adverse events over the 24-month controlled and open-label periods [4, 11]. Observational studies similarly documented strong persistence, with interruptions being infrequent and short-lived. The longest reported pause lasted 1.5 months due to gastrointestinal symptoms and headache, after which treatment was resumed [19].

Large real-world datasets confirmed these patterns. Pharmacovigilance and insurance-claims analyses reported adherence rates of 91.0–95.0%, with median values approaching 98.0–99.0% over 12 months and mean persistence of 326.2 ± 83.9 days in adult cohorts [46, 47].

## Discussion

In recent years, DMTs have dramatically changed the way clinicians manage SMA. It is although critical to differentiate between adult and pediatric patients, as treatment response and goals markedly differ between these groups. Although understanding these differences is crucial for selecting the right treatment and managing patient requests, RCTs have so far mainly focused on pediatric populations. Consequently, adult neurologists still lack the tools needed to set realistic expectations for new therapies and make informed and shared treatment decisions. While some benefit is generally expected, its specific nature cannot be precisely quantified based on RCT data alone. In this context, real-world studies offer an opportunity to fill this knowledge gap, despite their inherent limitations.

Regarding motor outcomes, when considering the adult population as a whole, risdiplam appears to promote stabilization or modest improvement of motor function, yet the magnitude of benefit seems to vary across age subgroups. Younger adults generally show greater responsiveness, likely reflecting higher residual motor neuron reserve. Conversely, older and more severely affected individuals tend to stabilize rather than improve, which remains clinically meaningful in a typically progressive condition. This pattern of response is similar to the one observed with nusinersen [3]. Accordingly, differences between phenotypes also emerge. Adults with SMA type 3, especially those who are still ambulant or retain antigravity upper-limb strength, are more likely to achieve measurable gains on motor scales, whereas those with SMA type 2 usually experience disease stabilization. Functional status plays a major role as well. Ambulatory adults may show detectable changes on HFMSE or RHS, while sitters and non-sitters often respond better on RULM or MFM-32, which are more sensitive to small improvements in upper-limb control. The heterogeneity of motor outcomes across studies highlights a fundamental challenge: current clinical instruments lack the sensitivity to detect subtle changes in motor function in adults with advanced disease. Future assessment may be improved by new clinical scales such as the Adapted Test for Neuromuscular Diseases (ATEND) scale [48], for which validation is currently ongoing, alongside insights from neurophysiological measures, offering more precise and standardized tools for evaluating even stable and slowly progressive adult populations.

Similar considerations apply to bulbar and respiratory function. Although evidence remains limited, available real-world data consistently suggest that risdiplam may improve swallowing and speech, even in individuals with advanced disease. Younger adults generally show greater responsiveness in bulbar domains, mirroring what is observed in motor outcomes. In cohorts of patients in their twenties or thirties, improvements were more frequently documented and often supported by significant reductions in SSQ scores. Phenotype and motor function appear to influence these responses. Individuals with SMA type 2—typically non-ambulant sitters or non-sitters—tend to present with more pronounced baseline bulbar weakness and are therefore more likely to experience noticeable and subjective improvement. Conversely, adults with SMA type 3, who usually remain ambulant or retain higher residual motor function, show milder baseline involvement and smaller measurable or subjective gains. The observation that clinically relevant improvement can occur even in severely affected, non-ambulant individuals, underscores the systemic reach of risdiplam and its potential effect on cranial motor neurons. In these more impaired patients, patient-reported instruments such as the SSQ or the bulbar items of the EK2 appear particularly sensitive to change, whereas clinician-rated scales like the FOIS or NdSSS may underestimate subtle but meaningful improvements.

Regarding respiratory function, most studies (including RCTs) still rely on FVC and FEV₁ measurements due to their wide availability and ease of use. Even with the small sample size in the adult cohorts and the short follow-up intervals, risdiplam appears to lead to at least a stabilization of respiratory outcome measures, showing trajectories that diverge from those observed in natural history data [49]. However, other outcome measures such as FEV1, Sniff Nasal Inspiratory Pressure (SNIP) [50], MIP, MEP, and Peak Cough Expiratory Flow (PCEF), together with nocturnal ventilation studies, may provide valuable complementary information for monitoring adult SMA patients. This is particularly relevant as spinal deformities and limited jaw opening—which are more common in adults than in children—can compromise the accuracy of conventional spirometric assessments. When used in conjunction with FVC and FEV₁, these measures may offer a more comprehensive picture of the respiratory function and further widen the possibility of longitudinal comparison.

As for patients’ treatment history, both treatment-naïve and nusinersen-experienced adults responded favorably, though in different ways. Switch cohorts typically maintained stability both in motor, bulbar and respiratory functions, suggesting that risdiplam can effectively preserve previously achieved gains. In treatment-naïve patients, improvements were more evident, depending on the SMA type and functional status, with several studies showing overall significant mean increases in motor scales.

The PROs, although heterogeneous, all indicate an overall improvement of the quality of life in adult patients, regardless of baseline motor function or the relative extent of measurable gain. Benefits have been reported in different domains, particularly in terms of reduced fatigability and pain, although the underlying mechanisms remain debated and may involve effects on the neuromuscular junction or dorsal root ganglia. These findings highlight a critical limitation in current SMA motor assessments: most standardized instruments fail to adequately capture fatigability, endurance, or subtle functional gains—except for the 6MWT, which obviously applies only to ambulatory individuals. This assessment gap may hinder the objective improvements in non-ambulatory patients, which nonetheless become evident through subjective reports. In this context, individualized functional scales, such as the Goal Attainment Scale (GAS) and the SMA Independence Scale (SMAIS), can provide valuable complementary information by reflecting patient-specific goals and perceived benefits, thus supporting a more patient-centered evaluation and strengthening the therapeutic alliance from both a clinical and psychological perspective. In this regard, the systematic collection of longitudinal, standardized, and SMA-specific PROs may help to control for the potential ‘new treatment effect’, particularly in adults who have long adapted to their lifelong condition.

Safety data are particularly informative. The incidence of AEs varies considerably across studies; however, the current literature indicates that the treatment is well tolerated in adult patients. Although the severity of adverse events according to the Common Terminology Criteria for Adverse Events (CTCAE) was not reported, the low rate of treatment discontinuation suggests that most adverse events were mild and transient—predominantly gastrointestinal and dermatological—and that overall tolerability was good. Notably, no serious SAEs were reported, except for the deaths of two SMA type 2 patients from aspiration pneumonia. In one case, reported by Iterbeke et al., the death occurred after 22 months of treatment and was considered unrelated to risdiplam. In the other case, reported by Sitas et al., the patient had been on risdiplam for six months and had anemia requiring periodic iron supplementation; however, further details were unavailable. Another consideration unique to the adult population is the potential risk of male infertility, a concern raised by non-clinical toxicologic findings in male germ cells of animals exposed to risdiplam [51]. Although a small case series of three patients suggests risdiplam is safe in this regard [52], and no other reports of infertility emerged from our review, a potential bias exists because formal fertility assessments were not conducted in the available studies. Definitive conclusions therefore require further research specifically designed to address this issue.

Finally, adherence to treatment is a crucial aspect of SMA management. A significant limitation of nusinersen is its invasiveness, which can lead to discontinuation due either to patient refusal or procedural constraints. This is particularly relevant in adults with SMA type 2, who often present with spinal deformities and severe motor limitations that complicate the intrathecal infusion. However, in the studies reviewed here, no definitive treatment discontinuations were reported, aside from the two deceased patients. Although varying follow-up durations and a lack of direct persistence data preclude definitive conclusions, the two pharmacovigilance studies retrieved confirm high adherence rates, thus constituting a strong point in favor of risdiplam's treatment profile.

## Limitations

Our study has several limitations. First, the available evidence is intrinsically weak, both because high-quality studies are scarce and because many reports do not clearly distinguish between adult and pediatric populations. Second, important prognostic and treatment-modifying factors—such as SMN2 copy number, concomitant therapies, and comorbid conditions—were frequently unreported. Third, substantial heterogeneity across studies in follow-up duration, baseline disease severity, and the clinical scales employed markedly limits the strength of the conclusions and prevents more powerful analyses, including meta-analytic approaches. Finally, the inherent constraints of real-world evidence, including the absence of control groups and the risk of selection bias, must be considered, as these issues complicate the attribution of observed outcomes solely to the effects of treatment.

## Conclusion

Risdiplam appears to be safe and generally well tolerated in adults with SMA, and current evidence suggests that it can stabilize disease progression and, in some cases, produce modest but clinically relevant improvements. Benefits have been reported across motor, bulbar, respiratory, and patient-reported domains, although with substantial variability related to SMA type and baseline conditions. Further focused and longitudinal research, using multidimensional outcome measures tailored to the patient’s current functional status, is needed to refine the role of risdiplam and to strengthen conclusions regarding its effectiveness in adults.

## Supplementary Information

Below is the link to the electronic supplementary material.

Supplementary file1 (DOCX 2751 KB)
