# Supplementary material for: Safety and effectiveness of risdiplam in adults with spinal muscular atrophy: a systematic review
Source: J Neurol. 2025 Dec 8;273(1):10. doi: 10.1007/s00415-025-13557-4 (PMC12682721; doi:10.1007/s00415-025-13557-4)
Supplement: Supplementary file 1 — Supplementary file1 (DOCX 2751 KB) [file 415_2025_13557_MOESM1_ESM.docx]

**Full search strings used:**

- **Pubmed:** (risdiplam[tiab]) AND (adult*[tiab] OR Adult[mh])
- **Scopus:** TITLE-ABS-KEY(risdiplam) AND TITLE-ABS-KEY(adult*)
- **Cochrane Library**: (risdiplam):ti,ab,kw AND (adult OR adults):ti,ab,kw
- [**ClinicalTrials.gov**](http://clinicaltrials.gov): risdiplam AND adult

**Supplementary Table 1. Quality assessment of included case series according to the Institute of Health Economics (IHE) Quality Appraisal Checklist for Case Series**

| **Title** | Risdiplam therapy in adults with 5q‐SMA: observational study on motor function and treatment satisfaction | Risdiplam in Adult Patients With 5q Spinal Muscular Atrophy: A Single-Center Longitudinal Study | Risdiplam: therapeutic effects and tolerability in a small cohort of 6 adult type 2 and type 3 SMA patients | Risdiplam improves subjective swallowing quality in non‐ambulatory adult patients with 5q‐spinal muscular atrophy despite advanced motor impairment | Risdiplam for the treatment of adults with spinal muscular atrophy: Experience of the Northern Ireland neuromuscular service | Risdiplam in non-sitter patients aged 16 years and older with 5q spinal muscular atrophy | Risdiplam Real World Data – Looking Beyond Motor Neurons and Motor Function Measures | Evaluation of risdiplam efficacy in 5q spinal muscular atrophy: A systematic comparison of electrophysiologic with clinical outcome measures | Two-year Risdiplam treatment in adults with spinal muscular atrophy: improvements in motor and respiratory function, quality of life and fatigue | Switching from Nusinersen to Risdiplam: A Croatian Real-World Experience on Effectiveness and Safety | Efficacy and safety of risdiplam in adults with 5q-associated spinal muscular atrophy: a nationwide observational cohort study in Austria |
| --- | --- | --- | --- | --- | --- | --- | --- | --- | --- | --- | --- |
| **Author** | Bjelica | Gavriilaki | Severa | Brakemeier | McCluskey | Ñungo Garzon | Sitas | Kessler | Iterbeke | Belančić | Keritam |
| **Year** | 2024 | 2024 | 2024 | 2023 | 2023 | 2023 | 2024 | 2023 | 2025 | 2024 | 2025 |
| **Was the hypothesis/aim/objective of the study clearly stated?** | Yes | Partial | Partial | Yes | Partial | Partial | Partial | Yes | Yes | Yes | Yes |
| **Was the study conducted prospectively?** | Yes | Yes | Yes | Yes | Unclear | Yes | Yes | Yes | Yes | No | Yes |
| **Were the cases collected in more than one centre?** | No | No | No | No | No | No | No | No | Yes | Yes | Yes |
| **Were patients recruited consecutively?** | Unclear | Unclear | Unclear | No | Unclear | Yes | Unclear | Yes | No | Unclear | Unclear |
| **Were the characteristics of the patients included in the study described?** | Yes | Yes | Yes | Yes | Yes | Yes | Yes | Yes | Yes | Yes | Yes |
| **Were the eligibility criteria for entry into the study clearly stated?** | Partial | Yes | No | No | Yes | No | Partial | Yes | Yes | Yes | Partial |
| **Did patients enter the study at a similar point in the disease?** | No | No | No | No | Partial | Partial | Partial | No | No | No | Yes |
| **Was the intervention of interest clearly described?** | Yes | Yes | Yes | Yes | Yes | Yes | Yes | Yes | Yes | Yes | Yes |
| **Were additional interventions (co-interventions) clearly described?** | Unclear | Unclear | Unclear | Unclear | No | Unclear | No | Unclear | Unclear | No | Yes |
| **Were relevant outcome measures established a priori?** | Yes | Yes | Yes | Yes | Yes | Yes | Yes | Yes | Yes | Yes | Yes |
| **Were outcome assessors blinded to the intervention that patients received?** | No | No | No | No | No | No | No | No | No | No | No |
| **Were the relevant outcomes measured using appropriate objective/subjective methods?** | Yes | Yes | Yes | Partial | Yes | Partial | Yes | Yes | Yes | Yes | Yes |
| **Were the relevant outcome measures made before and after the intervention?** | Yes | Yes | Yes | Yes | Yes | Yes | Yes | Yes | Yes | Yes | Yes |
| **Were the statistical tests used to assess the relevant outcomes appropriate?** | Yes | Yes | Yes | Partial | No | Yes | Yes | Yes | Yes | Yes | Yes |
| **Was follow-up long enough for important events and outcomes to occur?** | Yes | Yes | Partial | Partial | Partial | Partial | Partial | Partial | Yes | Yes | Yes |
| **Were losses to follow-up reported?** | Yes | Yes | Yes | Yes | Yes | Yes | Unclear | Yes | Yes | Yes | Yes |
| **Did the study provide estimates of random variability in the data analysis of relevant outcomes?** | No | Yes | No | Yes | No | No | Yes | Yes | Yes | Yes | Yes |
| **Were the adverse events reported?** | Yes | Yes | Yes | No | Yes | Yes | Yes | No | Yes | Yes | Yes |
| **Were the conclusions of the study supported by the results?** | Yes | Yes | Partial | Partial | Partial | Partial | Yes | Yes | Yes | Yes | Yes |
| **Were both competing interests and sources of support for the study reported?** | Yes | Yes | Yes | Yes | Unclear | Yes | Yes | Yes | Yes | Yes | Yes |
| **Total Score** | 27 | 29 | 23 | 22 | 20 | 25 | 26 | 29 | 32 | 30 | 35 |
| **Quality** | Moderate | Moderate | Moderate | Moderate | Moderate | Moderate | Moderate | Moderate | High | High | High |

**Supplementary Table 2. Quality assessment of included RCTs using the Cochrane Risk of Bias tool 2**

|  | Safety and efficacy of once-daily risdiplam in type 2 and non-ambulant type 3 spinal muscular atrophy (SUNFISH part 2): a phase 3, double-blind, randomised, placebo-controlled trial | Two-year efficacy and safety of risdiplam in patients with type 2 or non-ambulant type 3 spinal muscular atrophy (SMA) |
| --- | --- | --- |
| **Author** | Mercuri | Oskoui |
| **Year** | 2022 | 2024 |
| **D1** | Low | Low |
| **D2** | Low | Low |
| **D3** | Low | Low |
| **D4** | Low | Low |
| **D5** | Low | Low |
| **Overall** | Low risk of bias | Low risk of bias |

D1: Bias arising from the randomization process

D2: Bias due to deviations from intended intervention.

D3: Bias due to missing outcome data.

D4: Bias in measurement of the outcome.

D5: Bias in selection of the reported result.
